# Gelatinolytic activity in gingival crevicular fluid and saliva of growing patients with Marfan syndrome: a case-control study

**DOI:** 10.1186/s12903-019-0854-x

**Published:** 2019-07-24

**Authors:** Giuseppina Laganà, Giovanni Francesco Fasciglione, Andrea Biondi, Massimiliano Coletta, Giovanni Ruvolo, Paola Cozza

**Affiliations:** 1 Department of Clinical Sciences and Translational Medicine, University of Rome, Tor Vergata Via Montpellier 1, 00133 Rome, Italy; 2 Department of Cardiac Surgery Unit Chair Centre for Rare Diseases for Marfan Syndrome and Related Disorders, University of Tor Vergata General Hospital, Viale Oxford, 81 00133 Rome, Italy

**Keywords:** Marfan syndrome, Saliva, Crevicular fluid, Gelatinolytic activity, MMP

## Abstract

**Background:**

Aim of the study was to evaluate the gelatinolytic activity in the saliva and gingival crevicular fluid from a sample group of subjects with Marfan syndrome.

**Methods:**

Two groups were analyzed in this case-control study. A group of 28 subjects with Marfan syndrome (MG) was recruited from the Centre for Rare Disease, Marfan Clinic of Tor Vergata University Hospital. The second sample, 23 subjects, with the same characteristics and without any syndrome, was the control group (CG). Saliva and gingival crevicular fluid were collected and transferred to a sterile test tube and stored frozen at − 20 °C until analysis at the Medical Chemistry Laboratory. Gelatin substrate zymography was used for the evaluation and characterization of saliva and crevicular fluid proteinases. Correlation test and Student’s t-test have been used to analyze data.

**Results:**

In all samples different gelatin-degrading activities were observed. Two bands, which are related to the molecular weights of pro-MMP-9 and active MMP-9, respectively, were detectable in 100% of Marfan and control samples. MMP-2 activity was higher in Marfan group. Additional bands (55/48 kDa), corresponding to the activated forms of collagenase (MMP-13), were observed in saliva samples of both groups.

**Conclusions:**

The association of an enhanced activity by MMP-13 with an increased amount of active MMP-9 might be an important biomarker for the diagnosis of Marfan syndrome.

## Background

Marfan syndrome (MS) is a dominant hereditary connective tissue disease with a prevalence of 1–5/100,000 [1], but data are not always confirmed [2]. The mutations in the fibrillin-1 gene (*FBN1*) on chromosome 15 depend on alterations of the glycoprotein fibrillin-1, one of the major component of the microfibrils present in the connective tissue matrix [3]. These microfibrils are observed in the suspensory ligament of the lens, skeletal system, lungs, blood vessels, and skin [4]. Thus, MS is a systemic disease with individually different localization and degree of symptoms [5]. Cardiovascular complications, especially aortic dissection, are the major cause of morbidity and mortality [6, 7]. The varieties of musculoskeletal manifestations in Marfan syndrome concern the entire skeleton to various degrees, as it would be expected for a condition in which the pathology affects the basis of the building blocks of connective tissue and bone [8]. A number of oral manifestations, such as a high incidence of caries, tooth root deformity, abnormal pulp chambers with obliteration and a high susceptibility to periodontal pathologies, have been reported to be closely related to Marfan syndrome [9, 10]. Craniofacial alterations include dolichocephaly, maxillary and mandibular retrognathia and macrocephaly. Constriction of the maxilla and a high-arched palate are present, associated with crowding and posterior cross-bite and skeletal Class II malocclusion [11–13]. Functional alterations, such as oral breathing and Obstructive Sleep Apnoea Syndrome (OSAS)**,** are commonly observed [14].

The remodeling of the extracellular matrix, both in physiological and in pathological conditions, is a process finely regulated mainly by proteolytic enzymes, belonging to the MMPs family, characterized by the presence of a Zn^++^ in the active center [15, 16]. MMPs are synthesized and secreted as inactive precursors and their activation is a cascade process. Besides their natural substrates in the extracellular matrix, they are also able to degrade and modulate the biological activity of other molecules such as cytokines, growth factors and chemokines involved in periodontal diseases [17]. In oral diseases, a significant up-regulation of saliva levels of MMPs indicates that most extracellular matrix components undergo digestion to lower molecular weight forms [18]. MMPs are also involved in soft tissue remodeling which follows orthodontic tooth movement [19]. Notably, since fibrillin molecules are MMP substrates, MMPs catalysis disrupts fibrillin-rich microfibrils [20], the main actors being MMP-2, MMP-3 and MMP-13, constitutively produced by stromal cells or following an induction through growth factors. For this reason, these MMPs could be responsible for the physiological remodeling of the structure of fibrillin, while MMP-9 and MMP-12, which are major secretion products of macrophages, should be particularly important in the pathological turnover of fibrillin-rich microfibrils.

Many studies describe the use of saliva (S), gingival crevicular fluid (GCF) and oral mucosal transudates, to identify different oral and systematic diseases [21]. The collection of S and GCF is a minimally invasive procedure and the analysis of specific constituents provides a quantitative biochemical indicator for the evaluation of the local cellular metabolism indicating the personal periodontal health status [22]. Many pathological conditions have been largely studied using these biological liquids [23–29], but few studies in literature can be found about the saliva and crevicular fluid composition analysis as a diagnostic method for chronic or hereditary diseases. It is well known that mutations of FBN1 increased levels of circulating TGF-beta and abnormal regulation of matrix metalloproteinases (MMPs) are present in MS [15], but clinically acceptable risk stratification biomarkers have not been identified yet. No data are available with regard to the biochemical analysis of saliva and GCF in subjects with Marfan syndrome.

The purpose of this case-control investigation was to characterize the gelatinolytic activity of MMPs in saliva and crevicular fluid of MS patients, as compared with healthy control specimens.

## Methods

### Studied samples

A Marfan group (MG) of 28 subjects (17 Males, and 11 Females, mean age of 8.4 +/− 2.3 years), with a clinical and genetic diagnosis of Marfan syndrome was consecutively recruited, from February 2017 to November 2018, from the Centre for Rare Disease, Marfan Clinic of Tor Vergata University Hospital and evaluated in the Department of Orthodontics of the same University. The inclusion criteria of the Marfan group (MG) were: genetic assessment of Marfan syndrome, Caucasian ancestry, no previous orthodontic treatment, mixed stage of dentition, no post puberal stage (cervical stage CS5-CS6).

A second control group (CG), composes of 23 subjects (12 Males and 11 Females, mean age of 8.9 +/− 2.9 years), was recruited based on these inclusion criteria: Caucasian ancestry, no malocclusion, no previous orthodontic treatment, mixed stage of dentition, prepuberal vertebral maturation (no CS5-CS6). All the subjects in the control group were non-syndromic subjects.

Exclusion criteria for both MG and CG were: presence of gingival inflammation problems, periodontitis, dental caries, past or present orthodontic treatment, cleft lip and/or palate and other genetic diseases.

This project was approved by the Ethical Committee of the University of Rome Tor Vergata (Protocol number: 115/2017) and informed consent was obtained from the parents of each subject.

Collection of saliva and gingival crevicular fluid and elutions - To collect saliva (S) and gingival crevicular fluid (GCF) a 28 mm sterylized endodontic paper cone was used. The samples were collected with a different method: saliva was taken in the oral floor near minor salivar sublingual glands soaking the cone for 30 s, GCF was collected keeping the cone for 30 s into the gingival sulcus of left and right central incisors (3.1 and 4.1). To determine the reliability of the method one operator (A.B.) was trained in order to collect oral liquids in the same places. Saliva or GCF samples were then inserted into two different sterile test tubes and stored frozen at − 20 °C until analysis at the Department of Clinical Sciences and Translational Medicine, Medical Chemistry laboratory, University of Rome Tor Vergata. S and GCF were extracted by centrifugation at 9000 rpm for − 5 min in 50 μl of elution buffer (50 mM Tris-HCl, pH 7.5, 0.2 M NaCl, 5 mM CaCl_2_, and 0.08% Triton X-100). The elution procedure was repeated twice, and samples were stored at − 20 °C for further analysis.

#### Zymography

Gelatin substrate zymography was used for the evaluation and characterization of saliva and GCF proteinases in all the collected samples of saliva and GCF, as in Lombardi et al. [30]. Briefly samples [1 μg of proteins per lane; protein determination following Bradford [31]] were incubated for one hour at 37 °C with 5X solubilization buffer (0.25 M Tris, 0.8% sodium dodecyl sulphate (SDS), 10% glycerol and 0.05% bromophenolblue) and then run into a 12% SDS-PAGE gel containing 1 mg/ml of gelatin type B (Sigma Chemical Co.). After electrophoresis the gels were washed twice with a detergent buffer (2% Triton-X100 in water) in order to remove the SDS; subsequently**,** the gels are incubated at 37 °C for at least 22 h with activity buffer (50 mmol/L Tris-HCl, pH 7.5, 10 mmol/L CaCl2, 150 mmol/L NaCl) and then colored with Coomassie Blue R 250 (Bio-136 Rad, Hercules, CA) for 1 h and destained in water until you see the gelatinase activity bands, clear on a blue background. The intensities of the gelatinolytic activity areas were measured through an image analysis program (IMAGEQUANT TL; Amersham Biosciences, Piscataway, NJ) and quantified using a scale of arbitrary units (AU). The results obtained were analyzed using a statistical software (Prism 6 ver. 6.01, GraphPad Software). Correlation test and Student’s t-test were used to analyze the database. Data were expressed as mean ± SD.

Western immunoblots - Western blot experiments were performed, using antibodies against MMP-2 (M.W. 72–62 kDa) and/or MMP-9, molecular weight 92–82 kDa) to further characterize the bands seen by gelatin zymography analysis.

Western blotting was perfomed as in Lombardi et Al [30] using the same samples used in zymography (1 μg of proteins per lane; protein determination following Bradford [31]). Each sample was run in denaturing and reducing conditions in sample buffer (0.25 M Tris, 0.8% SDS, 10% glycerol and 0.05% bromophenol blue) on 12% SDS-polyacrylamide gel, after 2 min of boiling in presence of 2-mercaptoethanol. Gels were then transferred to nitrocellulose membrane (Amersham, Buckinghamshire, UK) in TRANSBLOT (Bio-Rad) using Towbin buffer (25 mM Tris, 192 mM glycine, and 20% methanol). Unspecific binding sites were blocked with 5% dry low-fat milk in phosphate buffered saline (PBS) for 1 h at 37 °C; membranes were then washed once with PBS and further incubated overnight at 4 °C with mouse monoclonal antibodies directed either against human gelatinase A (MMP-2) and/or human gelatinase B (MMP-9) (R&D Systems, Minneapolis, MN). Thereafter, three washes (5 min each) with PBS/Tween-20 (0.2%) were carried out, and membranes were then incubated with a 1/1000 dilution of horseradish peroxidase-labelled goat anti-mouse antibodies (Bio-Rad) in PBS for 2 h, followed by further washings (three washes, 5 min each) with PBS/Tween-20 (0.2%). The bands were visualized by using Enhanched Chemiluminescence (ECL) detection systems (Amersham U.K.).

## Results

**Fig. 1:**
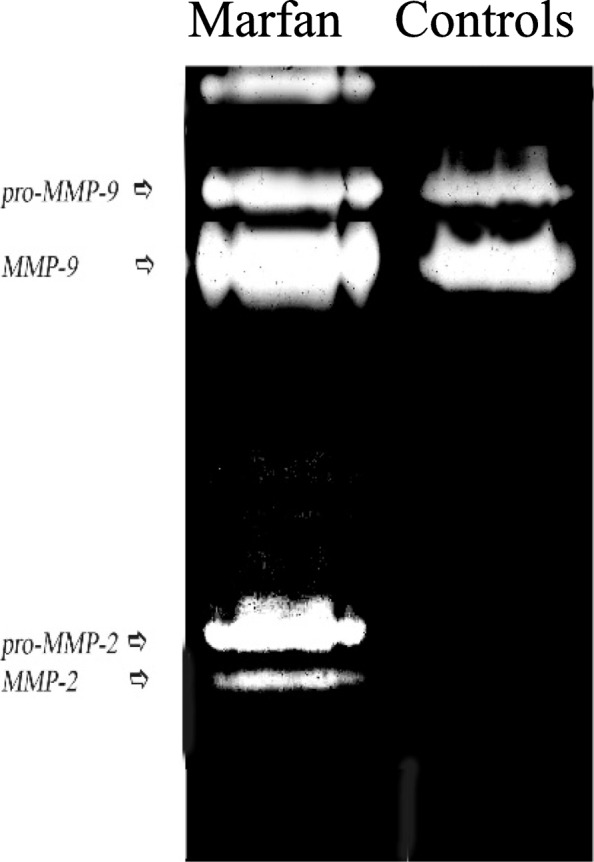
Gelatinolytic activity in GCF samples from Marfan patients and controls. Aliquots of a GFC sample were run on 12% polyacrilamide gel containing 1 mg/ml gelatin for gelatin zymography. Gels were incubated for 22 h at 37 °C to develop gelatinase activity

Figure 1 compares different gelatinolytic activities of GCF samples between the MG and CG. Two bands (i.e., 92 and 82 kDa, corresponding to the molecular weights of pro-MMP-9 and active MMP-9, respectively), were detectable in all GCF sample inspected (Table 1). On the other hand, though not present in all samples, in about 50% of the MG a lower MW activity (at 72 and 62 kDa, see Fig. 1) is detected (corresponding to pro-MMP-2 and active MMP-2, see Fig. 1), which is present in less than 30% of CG. No gelatinolytic activity (92, 82, 72 and 62 kDa) was observed if gels were incubated in a buffer containing MMP inhibitors (10 mM EDTA or 0.3 mM 1,10-phenanthroline), confirming that the observed activity was due exclusively to MMP presence (data not shown). Results obtained in Western blot confirmed the presence of the two forms of MMP-9 enzyme (pro- and active form) in all samples (Fig. 3a lane 1–2). Western blot experiments demonstrated the presence of MMP-2 (both forms) in the samples with activity at 72 and 62 kDa (Fig. 3b lane 1–2).

**Table 1:** Bands with gelatinolytic activity in GCF from Marfan patients and controls

*Marfan (n = 28)*			*Controls (n = 23)*		
*MMP*	*Detectable cases*	*Activity levels (au).* *Average ± SD*	*Detectable cases*	*Activity levels (au).* *Average ± SD*	*P* *(“t” Student’s test)*
proMMP-9	28 (100%)	395500 ± 36420	23 (100%)	199600 ± 36770	< 0.05
MMP-9	28 (100%)	1.171 × 10^6^ ± 223019	23 (100%)	632683 ± 163515	NS
proMMP-2	12 (44%)	1.219 × 10^6^ ± 219774	6 (28%)	133973 ± 97236	< 0.05
MMP-2	14 (50%)	579945 ± 83899	8 (33%)	663,2 ± 241,0	< 0.05

GCF, gingival crevicular fluid; MMP, matrix metalloproteinases; NS, not significant

Activity levels have been measured by an image analysis program (IMAGEQUANT TL; Amersham Bioscience, Piscataway, NJ). An arbitrary unit (au) scale was used. They have been analyzed by a stastical software(PRISM 6 ver. 6.01, GraphPad Software). Data were expressed as average ± standard deviation (SD)

The quantitative densitometric analysis of the bands reveals that bands at 92 kDa (pro-MMP-9), show a higher activity in the MG compared to the CG (395500 A.U. ± 36420 vs 199600 ± 36770; *P* <  0.05) (Table 1). Similar results were observed for the active form of MMP-9, which was more elevated in MG vs CG, even though in this last case the difference was less significant (1.171 × 10^6^ ± 223019 vs 632683 ± 163515; *P* > 0.05) (Table 1). For what it concerns MMP-2, as observed in Table 1, bands indicate increased activities in Marfan patients, as compared with the controls, and also these differences are significant (P <  0.05), with zymogenic forms predominating over the fully active enzyme (Table 1).

**Fig. 2:**
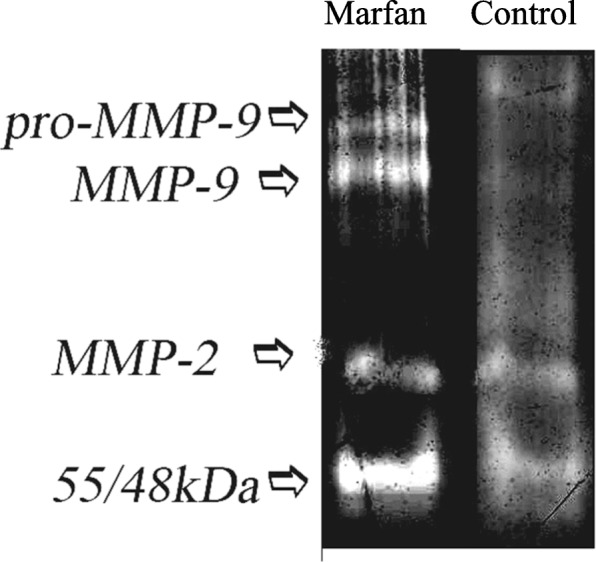
Gelatinolytic activity in saliva samples from Marfan patients and controls. Aliquots of saliva samples were run on 12% polyacrilamide gel containing 1 mg/ml gelatin for gelatin zymography. Gels were incubated for 22 h at 37 °C to develop gelatinase activity

**Fig. 3:**
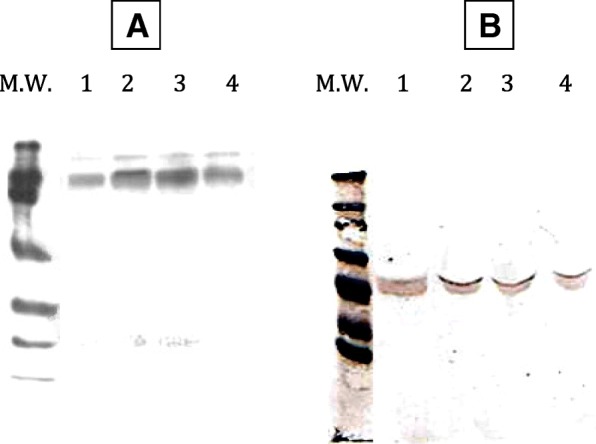
**a** Western blot obtained by using anti MMP-9 antibodies. Lane 1 GFC from CG; lane 2 GFC from Marfan group; lane 3 saliva from CG; lane 4 saliva from Marfan group. Bio-Rad Precision Plus Protein Western Standards were utilized. **b**. Western blot obtained by using anti MMP-2 antibodies. Lane 1 GFC from CG; lane 2 GFC from Marfan group; lane 3 saliva from CG; lane 4 saliva from Marfan group. Bio-Rad Precision Plus Protein Western Standards were utilized

Conversely, in saliva the two forms of MMP-9 (i.e., at 92 and 82 kDa, Fig. 2 and Fig. 3a lane 3–4) and of MMP-2 (i.e., at 72 and 62 kDa Fig. 2 and Fig. 3b lane 3–4) were detected but in this case no significant difference for the average activity can be detected between the active and non-active forms of gelatinases (Table 2). However**,** it is important to note that in saliva additional gelatinolytic bands, clearly different from pro- and active MMP-2 and/or MMP-9, were observed. In particular, the band, identified by the molecular weight of 48/55 kDa (active forms of MMP-13), appears in a substantial amount in most of samples from the MG.

**Table 2:** Bands with gelatinolytic activity in saliva from Marfan patients and controls

*Marfan (n = 28)*			*Controllo (n = 23)*		
*MMP*	*Detectable cases*	*Activity levels (au).* *Average ± SD*	*Detectable cases*	*Activity levels (au).* *Average ± SD*	*P* *(“t” Student’s test)*
proMMP-9	22 (78%)	15441 ± 2646	23 (100%)	11772 ± 2146	NS
MMP-9	13 (46%)	20752 ± 7322	9 (39%)	46307 ± 17506	NS
proMMP-2	16 (57%)	120629 ± 35599	13 (57%)	71236 ± 32762	NS
MMP-2	7 (25%)	7768 ± 2450	9 (39%)	10700 ± 5832	NS
48/55KDa	17 (61%)	105218 ± 16519	8 (61%)	59121 ± 14533	< 0,05

*MMP* matrix metalloproteinases, *NS* not significant

Activity levels have been measured by an image analysis program (IMAGEQUANT TL; Amersham Bioscience, Piscataway, NJ). An arbitrary unit (au) scale was used. They have been analyzed by a stastical software(PRISM 6 ver. 6.01, GraphPad Software). Data were expressed as average ± standard deviation (SD)

## Discussion

In Marfan patients an irregular microfibril morphology was observed reflecting incorporation of defective mutant allele products [32]. The oral manifestations of Marfan syndrome, though not specific, can be identified during a routine intraoral examination and may present a wide variation of commitments, similar to those found in the whole body. Although they are evaluated minor features for the diagnosis of the syndrome, oral changes are clinically important to select the treatment required to normalize the functions of chewing, breathing, phonation and swallowing [33].

Our experiments demonstrated the presence of gelatin activity in saliva and GCF, corresponding mainly to MMP-2 and MMP-9. In Marfan patients GCF a very high pro-MMP-9 levels were observed, correlated with MMP-9 activity, suggesting that a general inflammatory state could, most likely, be due to MMP-9 activity. Likewise, when MMP-2 activity was identified, this was more intense in the GCF from Marfan patients, indicating that this enzyme may be involved in soft tissue remodeling of dental processes. In saliva, there are no significant differences with respect to the average activity of MMP-9 and MMP-2 in both active and non-active forms. However, in several cases we observed additional bands at 55- and 48-kDa MW that could be the activated forms of collagenase 3 (MMP-13) [34].

MMP-13 is expressed by different cell types, such as fibroblasts, macrophages, osteoblasts, gingival epithelial cells and others. Normally, this collagenase is produced little or nothing in healthy adult tissues, but its expression increases dramatically in pathological situations where tissue repair or remodeling is necessary [34]. Although less abundant than gelatinases, MMP-13 may be involved in periodontal soft tissue degeneration. It was reported that in periodontitis patients, MMP-13 induces pro-MMP-9 activation in gingival tissue [35]. Therefore, this higher identifiable activity of MMP-13 in Marfan samples indeed could be associated to the degeneration of soft and hard tissues by pro-MMP-9 activation during progression of chronic periodontitis. Just because of its regulatory role it is easy to understand that even small changes in the collagenase-3 activity could lead to a significant degradation of the periodontal matrix and to an altered response in periodontal inflammations.

The observation of MMP-13 in the saliva of Marfan patients is a very novel observation and it opens a wider scenario both from the viewpoint of the disease mechanism and from that of the characterization of a potential biomarker, since MMP-13 is usually not expressed in normal adult tissue, but only whenever a tissue repair and remodelling is demanded.

The limit of this case-control study could be represented by the low number of subjects, due to a rare disease (Marfan syndrome). Moreover**,** this biochemical analysis can be considered the starting point for MMPs analysis; other MMPs could be involved in fibrillin pathological turnover and this hypothesis has to be demonstrated in future studies.

## Conclusions

Based on our study results it is clear that saliva and gingival crevicular fluid are useful indicators of MMPs activity and this study highlights a major activity of MMP-13 in subjects with Marfan syndrome**,** compared with healthy subjects. These results speculated a chronical inflammation state in the studied group. As well known [16] some members of the matrix metalloproteinase (MMP) family catabolize fibrillin molecules and disrupt fibrillin-rich microfibrils. MMP-2 and MMP-13 degrade fibrillin substrates, indicating that these enzymes could play an important role in the remodeling of physiological fibrillin. In view of its regulatory effects, it could be reasonable that slights changes in MMP-13 activity can induce significant alteration of periodontal matrix and changes in inflammatory response.

The association of an enhanced activity by MMP-13 with an increased amount of active MMP-9 is an important biomarker for the early diagnosis of Marfan syndrome, before genetic assessment. Further, this association identifies a potential therapeutic target for the amelioration of the progress in the Marfan syndrome.

## Data Availability

The datasets used and analysed during the current study are available from the corresponding author on reasonable request

## Abbreviations

AU: Arbitrary unit
CG: Control group
CS5: Cervical stage 5
ECM: Extracellular matrix
EDTA: Ethylenediaminetetraacetic acid
*FBN1*: Fibrillin-1 gene
GCF: Gingival crevicular fluid
MG: Marfan group
MMP: Matrix metalloproteinases
MS: Marfan Syndrome
PMSF: Phenylmethylsulphonyl fluoride
S: Saliva
SDS: Sodium dodecyl sulphate
SDS–PAGE: Sodium dodecyl sulphate–polyacrylamide gel electrophoresis

## References

[1] Groth KA, Hove H, Kyhl K, Folkestad L, Gaustadnes M, Vejlstrup N, Stochholm K, Ostergaard JR, Andersen NH, Gravholt CH (2015). Prevalence, incidence, and age at diagnosis in Marfan syndrome. Orphanet J Rare Dis.

[2] Stuart AG, Williams A (2007). Marfan’s syndrome and the heart. Arch Dis Child.

[3] Dietz HC, Cutting GR, Pyeritz RE, Maslen CL, Sakai LY, Corson GM, Puffenberger EG, Hamosh A, Nanthakumar EJ, Curristin SM (1991). Marfan syndrome caused by a recurrent de novo missense mutation in the fibrillin gene. Nature..

[4] Sakai LY, Keene DR, Engvall E (1986). Fibrillin, a new 350-kD glyco-protein, is a component of extracellular microfibrils. J Cell Biol.

[5] Rybczynski M, Bernhardt AM, Rehder U, Fuisting B, Meiss L, Voss U, Habermann C, Detter C, Robinson PN, Arslan-Kirchner M, Schmidtke J, Mir TS, Berger J, Meinertz T, Von Kodolitsch Y (2008). The spectrum of syndromes and manifestations in individuals screened for suspected Marfan syndrome. Am J Med Genet A.

[6] Ammash NM, Sundt TM, Connolly HM (2008). Marfan syndrome-diagnosis and management. Curr Probl Cardiol.

[7] Mattila KJ, Nieminen MS, Valtonen VV, Rasi VP, Kesäniemi YA, Syrjälä SL, Jungell PS, Isoluoma M, Hietaniemi K, Jokinen MJ (1989). Association between dental health and acute myocardial infarction. BM J.

[8] Avivi E, Arzi H, Paz L, Caspi I, Chechik A (2008). Skeletal manifestations of Marfan syndrome. Isr Med Assoc J.

[9] Hi H, Seo JB, Lee SH (2007). Imagine of Marfan syndrome: multisystemic manifestations. Radiographics..

[10] De Coster PJ, Martens LC, De Paepe A (2002). Oral manifestations of patients with Marfan syndrome: a case-control study. Oral Surg Oral Med Oral Pathol Oral Radiol Endod.

[11] Laganà G, Venza N, Paoloni V, Bertoldo F, Ruvolo G, Cozza P (2018). A 3D geometric morphometric analysis of the palatal morphology in Marfan’s syndrome. A preliminary study. J of Clin and Diagn Res.

[12] De Coster PJ, Martens LC, De Paepe A (2004). Orofacial manifestations of congenital fibrillin deficiency: pathogenesis and clinical diagnostics. Pediatr Dent.

[13] Gorlin RJ, Cohen MM, Levin LS (1990). Syndromes of head and neck.

[14] Paoloni V, Cretella Lombardo E, Placidi F, Ruvolo G, Cozza P, Laganà G (2018). Obstructive sleep apnea in children with Marfan syndrome: relationships between three-dimensional palatal morphology and apnea-hypopnea index. Int J Pediatr Otorhinolaryngol.

[15] Ramachandra CJ, Mehta A, Guo KW, Wong P, Tan JL, Shim W (2015). Molecular pathogenesis of Marfan syndrome. Int J Cardiol.

[16] Sbardella D, Fasciglione GF, Gioia M, Ciaccio C, Tundo GR, Marini S, Coletta M (2012). Human matrix metalloproteinases: an ubiquitarian class of enzymes involved in several pathological processes. Mol Asp Med.

[17] Cavalla F, Biguetti C, Jain S, Johnson C, Letra A, Garlet GP, Silva RM (2017). Proteomic profiling and differential messenger RNA expression correlate HSP27 and serpin family B member 1 to apical periodontitis outcomes. J Endod.

[18] Maciejczyk M, Pietrzykowska A, Zalewska A, Knaś A, Daniszewska I (2016). The significance of matrix metalloproteinases in oral diseases. Adv Clin Exp Med.

[19] Grant M, Wilson J, Rock P, Chapple I (2013). Induction of cytokines, MMP9, TIMPs, RANKL and OPG during orthodontic tooth movement. Eur J Orthod.

[20] Ashworth JL, Murphy G, Rock MJ, Sherratt MJ, Shapiro SD, Shuttleworth CA, Kielty CM (1999). Fibrillin degradation by matrix metalloproteinases: implications for connective tissue remodelling. Biochem J.

[21] Martí-Álamo S, Mancheño-Franch A, Marzal-Gamarra C, Carlos-Fabuel L (2012). Saliva as diagnostic fluid. Literature review, Oral Medicine and Pathology. J Clin Exp Dent.

[22] Champagne Catherine M. E., Buchanan William, Reddy Michael S., Preisser John S., Beck James D., Offenbacher Steven (2003). Potential for gingival crevice fluid measures as predictors of risk for periodontal diseases. Periodontology 2000.

[23] Choromańska K, Choromańska B, Dąbrowska E, Bączek W, Myśliwiec P, Dadan J, Zalewska A (2015). Saliva of obese patients - is it different?. Postepy Hig Med Dosw (Online).

[24] Kjölhede EA, Gustafsson PE, Gustafsson PA, Nelson N (2014). Overweight and obese children have lower cortisol levels than normal weight children. Acta Paediatr.

[25] Kidambi S, Raff H, Findling JW (2007). Limitations of nocturnal salivary cortisol and urine free cortisol in the diagnosis of mild Cushing’s syndrome. Eur J Endocrinol.

[26] Putignano P, Toja P, Dubini A, Pecori Giraldi F, Corsello SM, Cavagnini F (2003). Midnight salivary cortisol versus urinary free and midnight serum cortisol as screening tests for Cushing’s syndrome. J Clin Endocrinol Metab.

[27] Carda C, Mosquera-Lloreda N, Salom L, Gomez de Ferraris ME, Peydró A (2006). Structural and functional salivary disorders in type 2 diabetic patients. Med Oral Patol Oral Cir Bucal.

[28] Gupta G (2012). Gingival crevicular fluid as a periodontal diagnostic indicator- I: host derived enzymes and tissue breakdown products. J Med Life.

[29] De Aguiar MC, Perinetti G, Capelli jr J. The gingival crevicular fluid as a source of biomarkers to enhance efficiency of orthodontic and functional treatment of growing patients. Biomed Res. Int. 2017: Art.Id 3257235. 10.1155/2017/3257235 PMC5292379 28232938

[30] Lombardi F, Fasciglione GF, D’Apice MR, Vielle A, D’Adamo M, Sbraccia P, Marini S, Borgiani P, Coletta M, Novelli G (2008). Increased release and activity of matrix metalloproteinase-9 in patients with mandibuloacral dysplasia type a, a rare premature ageing syndrome. Clin Gent.

[31] Bradford MM (1976). A rapid and sensitive method for the quantitation of microgram quantities of protein utilizing the principle of protein-dye binding. Anal Biochem.

[32] Kielty CM, Raghunath M, Siracusa LD, Sherratt MJ, Peters R, Shuttleworth CA, Jimenez SA (1998). The tight skin mouse: demonstration of mutant fibrillin-1 production and assembly into abnormal microfibrils. J Cell Biol.

[33] Ferreira AEC Jr, Rodrigues Macedo LW, Lima Verde Quesado ME, Reboucas Diniz P. Marfan's syndrome: general informations and odontological manifestations. Oral Health and Dent Managem 2016;15-5: 329–331.

[34] Hernandez M, Valenzuela MA, Lopez-Otin C, Alvarez J, Lopez JM, Vernal R, Gamonal J (2006). Matrix metalloproteinase-13 is highly expressed in destructive periodontal disease activity. J Periodontol.

[35] Hernández M, Martínez B, Tejerina JM, Valenzuela MA, Gamonal J (2007). MMP-13 and TIMP-1 determinations in progressive chronic periodontitis. J Clin Periodontol.

